# The clinical efficacy of acupoint sticking combined with massage to treatment functional dyspepsia: A protocol of systematic review and meta-analysis

**DOI:** 10.1097/MD.0000000000023869

**Published:** 2020-12-18

**Authors:** Xiaoyan Wu, Weijun Jiang, Chunxiang Shi, Hui Qian, Xinguo Fan, Ping Zhou

**Affiliations:** GuangHua Hospital of Integrated Traditional Chinese and Western Medicine, Shanghai, China.

**Keywords:** acupoint sticking, functional dyspepsia, massage, protocol, systematic review

## Abstract

**Background:**

The objective of this meta-analysis was to summarize and identify the available evidence from studies to estimate the clinical value of acupoint sticking combined with massage (ASM) in the treatment of functional dyspepsia (FD), and provide clinicians with evidence on which to base their clinical decision making.

**Methods:**

This review will include all studies comparing clinical efficacy of ASM in the treatment of FD. The search strategy will be performed in 10 databases. We will not establish any limitations to language and publication status, published from inception to the August 2020. Two reviewers will screen, select studies, extract data, and assess quality independently. Outcome is alleviation of global dyspeptic symptoms, alleviation of individual dyspeptic symptoms, quality-of-life improvement, and safety. The methodological quality including the risk of bias of the included studies will be evaluated. We will carry out statistical analysis using RevMan 5.3 software.

**Results:**

This study will summarize current evidence to assess the efficacy and safety of ASM in the treatment of FD.

**Conclusion:**

The findings of this study will provide helpful evidence for the clinician, and will promote further studies, as well as studying the value of ASM.

**Registration number:**

INPLASY2020110072 (DOI number: 10.37766/inplasy2020.11.0072).

## 1 Introduction

Functional dyspepsia (FD) is a common and chronic upper gastrointestinal syndrome.^[1–3]^ According to the recently revised Rome IV criteria, FD is defined by Persistent or recurring dyspepsia for more than 3 months within the past 6 months, No demonstration of a possible organic cause of the symptoms on endoscopy, and No sign that the dyspepsia is relieved only by defecation or of an association with stool irregularities. This last criterion was introduced to rule out irritable bowel syndrome (IBS) as a possible cause of the symptoms, although around 30% of patients with FD also have IBS. There are 2 clinical syndromes, postprandial distress syndrome (PDS) and epigastric pain syndrome (EPS), although these can overlap, and additionally, belching, epigastric bloating, and nausea can occur in FD.

The peak age groups identified with FD is the 4th and 5th decades and uninvestigated dyspepsia affects around 21% of the population. The disease displays a periodic course, phases of slight or no symptoms alternating with periods of intensive complaints. Only 20% of patients with FD ever become free of symptoms in the long term. In the elderly, common gastrointestinal conditions can mimic FD, and therefore, in older patients, the syndrome may be more challenging to diagnose and treat, plus there are concurrent diseases and medication interactions that need to be considered.

There are several guideline-recommended pharmacotherapies for FD. However, due to the heterogeneous nature of the symptoms of FD, probably no single pharmacotherapy is able to manage the symptoms fully. Besides, individual symptoms may be caused by different mechanisms. As a result, the same drug may not be able to deal with the same symptom if it is originated from different mechanisms in different patients. In addition, despite being widely in use, prokinetic agents only demonstrate limited effectiveness.^[4–10]^

In the theory of traditional Chinese medicine (TCM), FD is termed as “distension and fullness” (in Chinese pinyin: Pi Man), “stomach pain” (in Chinese pinyin: Wei Wan Tong), and “retention” (in Chinese pinyin: Ji Zhi). TCM aims at harmonizing or improving the relationship between different organs and systems in the human body in order to relieve the symptoms of FD.^[11,12]^

For centuries, acupoint sticking combined with massage (ASM) has been used as a complementary and alternative medicine for western medicine in China. ASM is gaining popularity in other countries. Although ASM has been used clinically in the treatment of RA for many years, the efficacy and safety still need evidence-based medical research.^[13–16]^ To the best of our knowledge, there is no meta-analysis analysis the clinical efficacy of ASM for FD. Consequently, the objective of this meta-analysis was to summarize and identify the available evidence from these studies to estimate the clinical value of ASM, and provide clinicians with evidence on which to base their clinical decision making.

## 2 Methods

### 2.1 Study registry.

The protocol was registered on the International Platform of Registered Systematic Review and Meta-analysis Protocols (INPLASY2020110072). The preferred reporting items for systematic review and meta-analysis protocols (PRISMA) will serve as guidelines for reporting present review protocol and subsequent formal paper.^[17]^

### 2.2 Eligibility criteria for including studies

#### 2.2.1 Types of studies

We will include all studies comparing the clinical efficacy in the treatment of FD, including observational study and RCT. Any other types of studies, such as animal studies, case reports, case series, and review will all be excluded.

#### 2.2.2 Types of interventions

##### 2.2.2.1 Experimental group

All patients in the experimental group received ASM for their treatment in this study.

##### 2.2.2.2 Control group

The participants in the control group could receive any other treatments in this study.

#### 2.2.3 Types of patients

Patients suffered from FD will be included without sex, age, course, ethnicity, disease duration, or disease severity restrictions.

#### 2.2.4 Types of outcome measurements

**Primary outcomes**

(1) Alleviation of global dyspeptic symptoms, measured by global symptom improvement scale;
(2) Alleviation of individual dyspeptic symptoms (epigastric burning, epigastric pain, gastric emptying, or fullness), measured by individual symptom improvement scale.

**Secondary outcomes**

(1) Quality-of-life improvement;
(2) Safety

### 2.3 Literature sources and search.

A comprehensive literature search was conducted by searching both international and Chinese databases from their inception onwards to the October 2020. Databases included the MEDLINE, Springer, Web of Science, PubMed, EMBASE, the Cochrane Database of Systematic Reviews, Evidence Based Medicine Reviews, CNKI Chinese Biomedical Databases, Wanfang Digital Journals, China National Knowledge Infrastructure. The search terms were integrated as follows: ∗functional dyspepsia∗ AND ∗acupoint sticking combined ∗ AND ∗massage∗. No language restriction was set during the literature search.

### 2.4 Study selection

**Figure 1:**
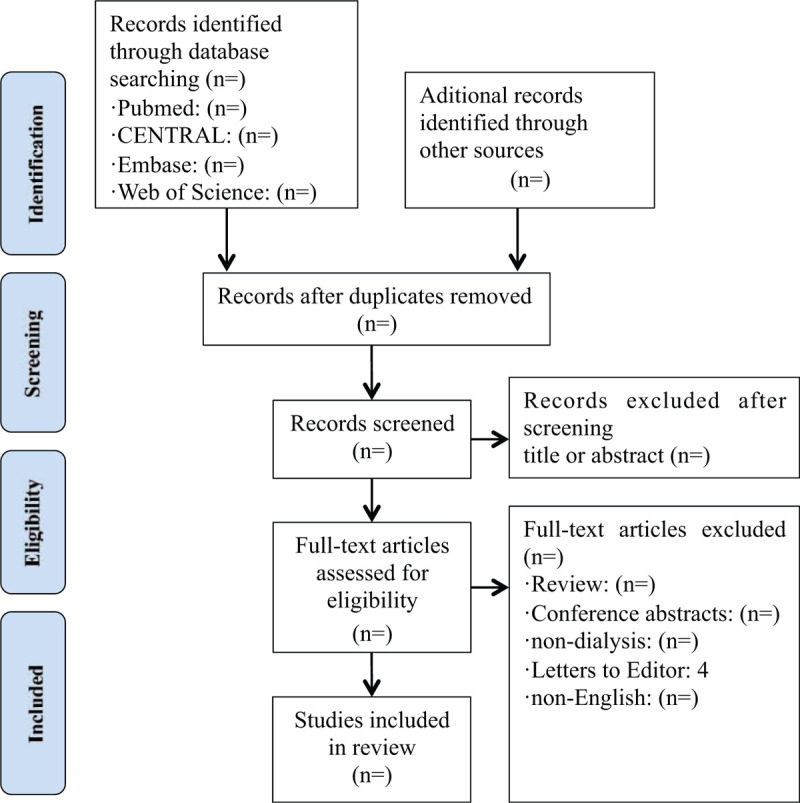
Study flow.

All duplicated studies will be imported into Endnote X7 software and excluded before the screening. Literature selection, data extraction, and methodological quality assessment were performed by 2 researchers independently. Disagreements were resolved by thorough discussions and consensus adjudication. A third reviewer would be consulted if disagreement could not be resolved, and his judgment was considered to be the final decision. The detail of the study selection will be presented in a PRISMA flow diagram (Fig. 1).

#### 2.4.1 Data extraction

Two authors will independently extract the following associated information from each included trial: first author, time of publication, sample size, randomization methods, blinding, concealment, allocation, details of intervention and controls, duration of follow-up, outcome measurement tools, and any other relevant information. A third senior author will help to reconcile any divergences between 2 authors.

#### 2.4.2 Quality assessment

The Cochrane risk of bias tool, which is recommended by the Cochrane Reviewer's Handbook 5.0.24, will be used to evaluate the quality of the included studies. Two independent reviewers will evaluate the quality of selected articles from the following 5 aspects: selection bias (random sequence generation or allocation concealment), performance bias and detection bias (blinding), attrition bias (incomplete outcome data), reporting bias (selective outcome reporting), and other biases. If necessary, we will contact the corresponding author to clarify issues. The result of the consistency evaluation will be presented with Kappa statistics, Kappa value <0.75 will be considered the consistency have reached. Any disagreements will be resolved through discussion or consultation.

#### 2.4.3 Subgroup analysis

We will preside over subgroup analysis to explore any potential heterogeneity and inconsistency based on the different factors.

#### 2.4.4 Sensitivity analysis

We will consider running sensitivity analysis to identify the robustness and stability of merged results by excluding studies with high risk of bias.

#### 2.4.5 Reporting bias

If necessary, we will examine the reporting bias using funnel plot and Egger regression test when >10 trials are included.

### 2.5 Data synthesis

The effectiveness of CHM treatments was assessed at SR level according to the Cochrane Handbook. No reanalyzing of the data using network meta-analysis approach was performed due to the insufficient number of trials sharing a common comparator, as well as head-to-head comparison between interventions. We extracted the pooled effect estimation from each meta-analysis. Pooled relative risk (RR) or odds ratio (OR) for dichotomous outcomes, and weighted mean difference (WMD) or standard mean difference (SMD) for continuous outcomes, accompanied by their respective 95% confidence intervals (CIs) were extracted as reported by the meta-analyses.

*I*-square (*I*^*2*^) values were also extracted for appraising heterogeneity among RCTs. The *I*^2^ value of <25%, 25% to 50%, >50% were regarded as an indicator for the presence of low, moderate, and high heterogeneity, respectively.

## 3 Discussion

FD is a common and chronic upper gastrointestinal syndrome. There is a lack of high-quality evidence on TCM in the treatment of FD patients. A recent meta-analysis studied that acupuncture probably has a positive effect on FD compared with usual care or passive therapies.^[18]^ However, how strong the effects of TCM are for FD patients is still unclear. We hope to provide more practical and targeted results to identify the therapeutic efficacy of ASM for FD patients in the current systematic review and meta-analysis.

The strength of this systematic review and meta-analysis will include search a comprehensive range of databases, including Chinese and English databases, more rigorous and detailed concerning quality assessment and data extraction. In addition, the findings obtained in the present study will provide helpful evidence in clinical practice. Furthermore, it will also help to promote further studies and clarify the direction for the future research. On the contrary, this study has several potential limitations. There may be a language bias, although there is not language limitation in this study. Moreover, there may be a large heterogeneity, which may bias the results.

## Author contributions

XYW, CXS, HQ, WJJ, and XGF conducted the protocol and drafted the manuscript. All authors participated in the design of the study. XYW and WJJ are co-first authors. PZ is co-corresponding author of this manuscript. All authors read and approved the final manuscript.

**Conceptualization:** Chunxiang Shi, Xinguo Fan.

**Data curation:** Xiaoyan Wu, Chunxiang Shi.

**Formal analysis:** Xiaoyan Wu, Weijun Jiang.

**Funding acquisition:** Weijun Jiang.

**Investigation:** Hui Qian.

**Methodology:** Weijun Jiang.

**Project administration:** Ping Zhou.

**Supervision:** Ping Zhou.

**Validation:** Xinguo Fan.

**Visualization:** Xinguo Fan.
